# Long-term observation of mortality among inpatients evacuated from psychiatric hospitals in Fukushima prefecture following the Fukushima nuclear disaster

**DOI:** 10.1038/s41598-021-94152-1

**Published:** 2021-07-19

**Authors:** Toshihiro Terui, Yasuto Kunii, Hiroshi Hoshino, Takeyasu Kakamu, Tomoo Hidaka, Tetsuhito Fukushima, Nobuo Anzai, Daisuke Gotoh, Itaru Miura, Hirooki Yabe

**Affiliations:** 1 Department of Neuropsychiatry, Fukushima Medical University School of Medicine, Hikarigaoka-1, Fukushima, 960-1295 Japan; 2 Department of Disaster Psychiatry, International Research Institute of Disaster Science, Tohoku University, 2-1 Seiryo-machi, Aoba-ku, Sendai, 980-8573 Japan; 3 Department of Hygiene and Preventive Medicine, Fukushima Medical University School of Medicine, Hikarigaoka-1, Fukushima, 960-1295 Japan; 4 Graduate School of Clinical Psychology, Teikyo Heisei University, 2-51-4 Higashi-Ikebukuro, Toshima-Ku, Tokyo, 170-8445 Japan

**Keywords:** Natural hazards, Medical research

## Abstract

The debate regarding the need for hospital evacuation and the evacuation distance remains rather chaotic. Furthermore, the relationship between hospital evacuation and the prognoses of psychiatric inpatients has not yet been investigated. We aimed to reveal the association between the long-term prognosis of psychiatric inpatients evacuated immediately following the Fukushima Daiichi Nuclear Power Plant accident and their backgrounds. In this retrospective cohort study, 777 psychiatric inpatients who were immediately evacuated from their hospitals following the accident were included for analysis. Survival time was the primary outcome. We conducted univariable and multivariable analyses to examine the associations between mortality and linear distance of evacuation and different backgrounds, including psychiatric/physical traits. Univariable analysis showed that the estimated survival time among patients was significantly associated with their evacuation distance. A multivariable analysis showed that a longer evacuation distance had a significantly lower hazard ratio (HR) and resulted in lower mortality. In contrast, older patients with physical complications of respiratory disease (International Statistical Classification of Diseases and Related Health Problems 10th revision, J00–99) and genitourinary disease (N00–99) showed a significantly higher HR and had a higher mortality than patients without these complications. To prevent death among elderly psychiatric inpatients with physical comorbidities during disasters, the evacuation destination should be determined taking into consideration the evacuees’ tolerance for long-distance transportation and the availability of post-evacuation care in the destination hospitals.

## Introduction

Hospital evacuation following the occurrence of a massive natural disaster has been controversial for a long time. Professionals have been discussing whether inpatients should be evacuated or not in consideration of their longitudinal outcomes, such as mortality after evacuation. Although there are some disasters where evacuation-related deaths did not occur^1^, evacuation is known to be associated with increased mortality in patients and nursing facility residents^2–4^. Moreover, since sheltering-in-place (no evacuation) was also found to elevate mortality risk, this has added further controversy to the discussion^5^. Additionally, Nomura et al.^2^ verified the influence of evacuation distance on mortality among residents of nursing homes and showed that it had no meaningful effect. Evacuation distance may be a factor in the prognosis of evacuees because the physical condition of patients cannot always be adequately controlled during evacuation. However, there has been little opportunity to assess this issue. In light of these arguments, medical professionals must triage patients and decide whether they should be evacuated (and where they should be evacuated) based on their own instincts or experiences^6–8^.

There have been few activity reports or opportunities for debate regarding the hospital evacuation of psychiatric patients related to massive disasters^9,10^. Villami–Salcedo et al. referred to the “diversity” of psychiatric inpatients^11^. They suggested that bedridden patients with comorbidities should be prioritized for evacuation, while those with major depressive or personality disorders could still be offered support. However, they argued that the evacuation of psychiatric hospital patients is based on the patients’ health conditions, which were prioritized. Nevertheless, no study has identified such a characteristic population’s long-term prognosis following hospital evacuation with practical triage, especially for deciding evacuation destination/distance. Moreover, evacuees’ psychiatric/physical factors, which might be related to the increase in mortality, have not been estimated. As the mortality of psychiatric patients is higher than that of the general population^12–14^, additional efforts to reveal the mortality after evacuation are necessary for disaster medicine practitioners to address psychiatric patients as a “disaster-vulnerable” population and offer disaster medical support in order to prevent disaster deaths among them.

**Figure 1:**
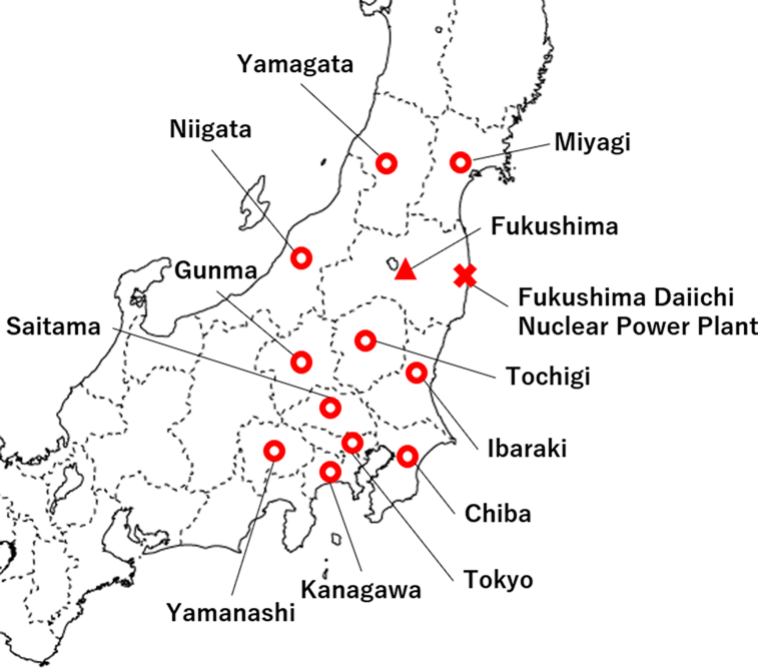
A map of prefectures where destination hospitals for psychiatric patients evacuated because of the FDNPP accident are located. Destinations are shown as circles. The triangle shows Fukushima prefecture, which includes the hospitals where evacuated psychiatric inpatients had been hospitalized before the GEJE and FDNPP. The cross shows the FDNPP. Microsoft PowerPoint (https://www.microsoft.com/en-us/microsoft-365/powerpoint) version 2016 was used to create the map.

The Great East Japan Earthquake (GEJE) and the subsequent Fukushima Daiichi Nuclear Power Plant (FDNPP) accident in 2011 obliged inpatients in the Soso area (including psychiatric patients) to be evacuated from hospitals where they were admitted to other hospitals within or outside Fukushima prefecture (Table 1)^15–19^. As with previous disasters^6,8^, with radiological screening, some bedridden inpatients were triaged by the Disaster Medical Assistance Team (DMAT) and other teams to select transportation methods or destinations in consideration of the patient’s physical condition. Patients who were physically unstable and patients who had difficulty tolerating long-distance evacuation were evacuated to local hospitals^17^. In fact, Gotoh et al. found that psychiatric inpatients who had been evacuated to hospitals inside Fukushima prefecture had higher mortality in 2011 than those evacuated out of the region (Fig. 1)^18^. In Japan, contrary to most other developed countries, efforts to provide community-based care for psychiatric patients have been very limited; therefore, many psychiatric patients still receive hospital care by prolonged hospitalization for a long time^20^. Moreover, the aging of psychiatric patients has led to a growing demand for the treatment of physical medical conditions in psychiatric hospital care^21^. By examining this population of evacuees prospectively, we may be able to describe how practitioners performed triage at that time and shed light on an overlooked or technically difficult point to resolve―the evacuation of psychiatric hospital inpatients and evacuation distance. Such discussion should be essential, especially for Japan, which is a country with many elderly psychiatric inpatients with physical conditions, for planning in cases of complicated disasters in the future.

**Table 1:** Timeline of the Fukushima Nuclear Disaster and associated hospital evacuation in 2011.

Date	Time	Event
March 11	02:46 PM	A magnitude 9.0 earthquake struck Japan A total of 11 units of the Fukushima Daiichi (and Daini) Nuclear Power Plant (FDNPP) automatically shut down
	07:03 PM	"Declaration of a Nuclear Emergency Situation" at the FDNPP was announced by the Japanese government
	09:23 PM	Residents within a 3 km radius of the FDNPP were ordered to evacuate
March 12	05:44 AM	The evacuation zone was expanded to a radius of 10 km **The evacuation of some psychiatric hospital inpatients began**
	03:36 PM	A hydrogen explosion occurred at the FDNPP Unit 1
	07:04 PM	The evacuation zone was expanded to a radius of 20 km
March 14	11:01 AM	A hydrogen explosion occurred at the FDNPP Unit 3
March 15	06:10 AM	The sound of an explosion resonated at the FDNPP Unit 2
	09:40 PM	A fire broke out at the FDNPP Unit 4 Residents within a 20–30 km radius of the FDNPP were ordered to evacuate indoors

The Sendai Framework for Disaster Risk Reduction (2015–2030) includes “reduce global disaster mortality” as one of its seven global targets. In accordance with the recent view that the common cause of hospital evacuation is natural disasters^22^, identifying predictors of or factors associated with mortality after evacuation may allow us to achieve this agenda through policy proposals or practical strategies.

The purpose of this study was to elucidate the association between long-term survival of psychiatric inpatients who were evacuated and their evacuation distance and background factors (especially psychiatric diagnoses and physical comorbidities).

## Methods

In this retrospective cohort study, the target population was psychiatric patients in Fukushima prefecture at the time of the GEJE who had been evacuated immediately after the FDNPP accident and whom the Fukushima prefectural government had registered in the Matching Project for Community Transition. This project began in July 2013 and sought to provide evacuated psychiatric inpatients with government support for their post-evacuation return to the Fukushima prefecture and community transition after the GEJE. Since 2012, project staff had been approached to register evacuated patients in the hospital where they had been admitted to at the time of the GEJE and at the beginning of the survey. Information regarding the evacuation hospital of patients, psychiatric diagnosis, and physical complications was collected by staff during visits and by survey slips to hospitals. Furthermore, registered patients were followed up yearly with a status survey. A total of 789 patients were registered in this project. Six patients who had been evacuated from their home to hospitals outside Fukushima prefecture were registered in response to the request of evacuated hospitals but were excluded from the analysis. We also excluded two who had unclear clinical outcomes (survival or death) and four whose survival time could not be calculated; therefore, 777 patients were included in the analysis.

Survival time was the main outcome of this study. All patients were traced from March 11, 2011 to death or the end of the study period (July 31, 2019). Most patients who were traced had been hospitalized (in destination hospitals, hospitals in Fukushima prefecture where they returned by the Matching Project, and other hospitals where they were transferred to after the evacuation). One reason for follow-up discontinuity was community transition (discharged to their original house or facilities that were intended to provide community-based care to patients [e.g., group home, nursing home]). Additionally, the follow-up was discontinued for some of the patients because of returning to the hospital they were admitted to before the accident.

We calculated the linear distance between hospitals where patients were originally hospitalized at the time of the disaster and evacuation destination hospitals, which was the exposure variable of this study. Our hypothesis stipulated that a longer evacuation distance was associated with higher post-evacuation mortality. Other items included as covariates were the patient’s age on March 11, 2011, sex, psychiatric diagnosis, and physical complications.

We classified inpatients’ psychiatric diagnoses and physical complications based on the International Statistical Classification of Diseases and Related Health Problems 10th revision (ICD–10), which had been internationally adopted as the standard classification for physical and mental health conditions. A previous study that focused on psychiatric patients’ mortality also applied the ICD for classifying diagnoses^23^. F and some G codes in the ICD–10 were adopted for the classification of psychiatric diagnosis: organic, including symptomatic, mental disorders (F00–09); mental and behavioral disorders due to psychoactive substance use (F10–19); schizophrenia and schizotypal and delusional disorders (F20–29); mood (affective) disorders (F30–39); neurotic, stress-related, and somatoform disorders (F40–48); behavioral syndromes associated with physiological disturbances and physical factors (F50–59); disorders of adult personality and behavior (F60–69); mental retardation (F70–79); disorders of psychological development (F80–89); and epilepsy (G40–41). We also classified physical complications according to the ICD-10 as follows: certain infectious and parasitic diseases (A00–B99); neoplasms (C00–D48); diseases of the blood and blood-forming organs and certain disorders involving the immune mechanism (D50–89); endocrine, nutritional and metabolic diseases (E00–90); diseases of the nervous system (G00-99, but not G40–41); diseases of the eye and adnexa (H00–59); diseases of the ear and mastoid process (H60–95); diseases of the circulatory system (I00–95); diseases of the respiratory system (J00–99); diseases of the digestive system (K00–93); diseases of the skin and subcutaneous tissue (L00–99); diseases of the musculoskeletal system and connective tissue (M00–99); diseases of the genitourinary system (N00–99); and injury, poisoning, and certain other consequences of external causes (S00–T98).

We performed univariable analysis to estimate the association between inpatients’ survival time and evacuation distance and each of the covariates using the log-rank test. On confirming factors to be significantly associated with the survival time on univariable analysis, a multivariable analysis was conducted by submitting these factors and evacuation distance and by adopting the Cox proportional hazards regression analysis. SPSS version 25 (IBM Corp, Armonk, NY, USA) was used for the analyses, and R was used to compute the linear distance between the hospitals. All research was performed in accordance with Declaration of Helsinki—Ethical Principles for Medical Research Involving Human Subjects. This study was approved by the Ethics Committee of Fukushima Medical University (No. 29389). As the study required contact with the Fukushima prefecture and the data of all registered patients were anonymized, the requirement for written informed consent was waived by the committee.

## Results

### Demographic characteristics of participants and the univariable analysis

The basic characteristics of the patients are shown in Table 2. The calculated median linear distance for evacuation among the entire population was 169 km (interquartile range [IQR] 98–226 km). The median age of the patients in 2011 was 66 years (IQR 56–76 years). Over half of the patients’ psychiatric diagnoses were classified as F20–29 (57.1%).

**Table 2:** Results of the univariable analysis for the association between mortality and basic characteristics of participants (N = 777).

	n (%) or Median (IQR)	*p* value
**Linear distance for evacuation, km**		
Median (IQR)	169 (98–226)	< 0.001***^†^
**Sex**		
Male	390 (50.2)	0.391
Female	387 (49.8)	
**Age, years**		
Median (IQR)	66 (56–76)	< 0.001***^‡^
**Psychiatric diagnosis**		
F00-09. Organic, including symptomatic, mental disorders	205 (27.1)	< 0.001***^‡^
F10-19. Mental and behavioral disorders due to psychoactive substance use	38 (5.0)	0.027*^†^
F20-29. Schizophrenia, schizotypal, and delusional disorders	432 (57.1)	< 0.001***^†^
F30-39. Mood (affective) disorders	57 (7.5)	0.505
F40-48. Neurotic, stress-related, and somatoform disorders	10 (1.3)	0.962
F50-59. Behavioral syndromes associated with physiological disturbances and physical factors	0 (0.0)	
F60-69. Disorders of adult personality and behavior	2 (0.3)	0.653
F70-79. Mental retardation	78 (10.3)	0.039*^†^
F80-89. Disorders of psychological development	3 (0.4)	0.637
G40-41. Epilepsy	36 (4.8)	0.530
**Physical complication**		
A00-B99. Certain infectious and parasitic diseases	15 (1.9)	0.949
C00-D48. Neoplasms	23 (3.0)	0.602
D50-89. Diseases of the blood and blood-forming organs and certain disorders involving the immune mechanism	8 (1.0)	0.356
E00-90. Endocrine, nutritional, and metabolic diseases	100 (12.9)	0.783
G00-99. Diseases of the nervous system (except G40-41, epilepsy)	45 (5.8)	0.366
H00-59. Diseases of the eye and adnexa	13 (1.7)	0.383
H60-95. Diseases of the ear and mastoid process	3 (0.4)	0.954
I00-95. Diseases of the circulatory system	83 (10.7)	0.281
J00-99. Diseases of the respiratory system	36 (4.6)	< 0.001***^‡^
K00-93. Diseases of the digestive system	54 (6.9)	0.385
L00-99. Diseases of the skin and subcutaneous tissue	7 (0.9)	0.560
M00-99. Diseases of the musculoskeletal system and connective tissue	12 (1.5)	0.580
N00-99. Diseases of the genitourinary system	14 (1.8)	< 0.001***^‡^
S00-T98. Injury, poisoning, and certain other consequences of external causes	13 (1.7)	0.060

IQR: interquartile range.

The log-rank test was adopted for the analysis.

The mortality rates of participants with each psychiatric diagnosis or physical complication were compared with those of patients unaffected by any disease.

*p < 0.05, **p < 0.005, ***p < 0.001.

^†^Factor showed a tendency toward lower mortality.

^‡^Factor showed a tendency toward higher mortality.

The median survival time among the evacuated inpatients was 1014 days (IQR 153–2703 days). The patients' estimated 1-, 3-, and 5-year survival probabilities after March 11, 2011 were 87.3%, 74.3%, and 65.0%, respectively. According to the univariable analysis with the log-rank test, the factors associated with a longer survival time and a lower mortality were longer linear distance for evacuation (*p* < 0.001) and psychiatric diagnoses classified as F10–19 (*p* = 0.027), F20–29 (*p* < 0.001), or F70–79 (*p* = 0.039). Conversely, factors that were associated with a shorter survival time and higher mortality were the patients’ age (*p* < 0.001), psychiatric diagnoses classified as F00–09 (*p* < 0.001), and physical complications classified as J00–99 (*p* < 0.001) and N00–99 (*p* < 0.001).

### Multivariable analysis of participants’ characteristics

Items that were significant in the former analysis were entered into the multivariable analysis; these results are shown in Table 3. The linear distance for evacuation remained significantly associated with survival time and was associated with a lower mortality (HR 0.997; 95% confidence interval [CI] 0.996–0.999). Conversely, the factors that remained significantly associated with a shorter survival time and a higher mortality included the patients’ age (HR 1.065; 95% CI 1.051–1.079) and physical complications classified as J00–99 (HR 3.635; 95% CI 2.423–5.453) and N00–99 (HR 3.248; 95% CI 1.784–5.913).

**Table 3:** Results of the multivariable analysis for the association between mortality and characteristics of participants.

	HR	95% CI	p-value
Linear distance for evacuation	0.997	0.996–0.999	0.002**
Age	1.065	1.051–1.079	< 0.001***
**Psychiatric diagnosis**			
F00–09	0.848	0.567–1.268	0.421
F10–19	0.361	0.130–1.003	0.051
F20–29	0.726	0.498–1.058	0.096
F70–79	0.918	0.556–1.516	0.738
**Physical complication**			
J00–99	3.635	2.423–5.453	< 0.001***
N00–99	3.248	1.784–5.913	< 0.001***

The Cox proportional hazards regression model was adopted for the analysis.

Mortality rates of participants with each psychiatric diagnosis or physical complication were compared with those of patients unaffected by any disease.

HR: hazard ratio, CI: confidence interval.

*p < 0.05, **p < 0.005, ***p < 0.001.

## Discussion

This is the first study to assess the association between post-evacuation mortality and evacuation distance in psychiatric hospital inpatients following a massive disaster. After adjusting for the covariates, which were significantly associated with the prognoses, a longer evacuation distance was associated with a significantly lower risk of mortality. This result could prompt us to probe in greater depth patient triage by medical staff at that time, and the post-evacuation care.

Medical triage for hospital evacuation appears to have initially focused on the binary decision of whether to evacuate or whether to shelter-in-place. As mentioned above, while previous reports have stated that patients’ evacuation was associated with an increased risk of death^2–4^, there is also evidence that sheltering-in-place increased the risk of death in some evacuated inpatients^5^. Unexpected hospital evacuation can make arranging for proper transportation and evacuee care at the evacuation site difficult, which may increase its effect on the mortality^4^. Conversely, Shimada et al.^5^ found that the adverse effect of staying in hospitals on the mortality appeared to be related to the severe environment of hospitals (e.g., lack of a heating system and power generator, food shortage, and lack of medical staff) during and after the incident.

Additionally, the current study may provide insight into triage for decision-making based on the evacuation distance and destination. We hypothesized that a longer evacuation distance would be associated with a higher mortality owing to the vulnerable evacuees’ longer exposure to unsteady transportation and cold climate^5^. Respiratory and genitourinary diseases, both of which are reported to increase during natural disasters, including the GEJE^24,25^, were associated with the patients’ long-term prognoses in this study. Both climate (cold environment’s effect on pneumonia)^26^ and medical equipment (delay of dialysis provision)^27^ complicate controlling for these diseases. However, in contrast to this hypothesis, we discovered that a longer evacuation distance was significantly associated with lower mortality. Although some patients’ background information, including physical complications, was adjusted, it could not reflect the physical emergency level completely. Thus, we consider that our results may be attributed to the medical professionals’ qualified triage in assessing patients’ evacuation destination based on their unmeasurable, more practical physical assessments. Namely, the medical staff may have made decisions on the evacuation destination based on whether the patients were physically stable and able to tolerate long-distance evacuation, or vulnerable and less able to endure long-distance travel.

In addition to the main analysis, we confirmed that there was a significant difference in mortality between inpatients evacuated to hospitals within and outside Fukushima prefecture in the optional log-rank test; the discrepancy was seen soon after March 11, 2011 (Supplementary Fig. S1). In light of such a discrepancy, conceivable physical triage at that time (shorter evacuation distance for vulnerable patients) and factors significantly associated with mortality such as patients’ age and physical conditions, we should consider whether the hospitals in Fukushima prefecture that received the evacuees were adequately prepared to receive the patients, although we do not know what type of care was provided in the evacuation destination hospitals.

Nomura et al.^3^ discussed the effect of unplanned hospital evacuations on the increasing mortality risk considering the evacuation sites’ preparedness for evacuee care. Not only hospitals in Hama-dori, where FDNPP was located, but also those in other regions in Fukushima prefecture were afflicted by the earthquake. For example, seven of the eight disaster center hospitals were partially damaged by the earthquake, and more than half of them had to restrict the admission of inpatients immediately following the incident^28^. Furthermore, less than half of the hospitals, not limited to the disaster center hospitals, were able to continue normal operations following the earthquake^29^. Given that almost half of the inpatients admitted to the psychiatric wards in Japan were over 65 years of age^30^, providing them with disaster emergency support is not limited to evacuation transportation and management of their physical medical conditions is an urgent issue.

In light of these results, both the tolerance of acute hospital transportation and the necessity for post-evacuation medical care need to be considered to minimize the risk of mortality related to unintended hospital evacuation, by determining appropriate destination hospitals.

After the GEJE, the Disaster Psychiatric Assistance Team (DPAT) was established in 2013 and has provided professional support to medical facilities (e.g., support of patients’ evacuation) as one of their activities^31,32^. Moreover, during the acute phase of disasters, DPAT members have also used the Simple Triage And Rapid Treatment (START) Adult Triage method for brief assessment of sufferers, similar to other disaster support teams (e.g., DMAT). Continuous training among medical staff for necessary and adequate assessment of the physical status, sharing information, and cooperation with other teams or destination hospitals is important to decrease preventable disaster deaths among elderly psychiatric inpatients with a variety of physical complications.

This study has several limitations. The most important limitation is that each patient’s evacuation transportation method and post-evacuation care, which were both important confounding factors, were not recorded. There could have been instances of inappropriate means of medical transportation to maintain the patients’ physical condition, which we could not detect. With regard to health status during evacuation, accurate information pertaining to the frequency of transportation of each evacuee could not be gathered. In addition, considering patients’ post-evacuation care and the medical equipment available at destination hospitals might also determine patients’ prognosis. A second important limitation is that censoring might underestimate the effects of some psychiatric disorders on mortality. Recent reports identified specific psychiatric diseases or symptoms as important risk factors for suicide after natural disasters^33^. Moreover, it is also important to consider that old age is one of the risk factors for post-disaster suicide^33^. The target population in this study included patients who had achieved community transitions and might have become somewhat independent from medical practitioners. In addition to this concern, most patients had been hospitalized during the follow-up period; thus, our results may only be relevant to patients receiving long-term care. Therefore, future studies should follow-up with evacuees not only during admissions or until death, but also after discharge and return to the community. Third, the registered patients’ physical complications were collected from practitioners’ interviews and survey slips after the disaster; details regarding onset and severity, which could determine participants’ long-term prognosis, are unclear. Furthermore, other health status scales, which reflect patients’ emergency levels more clearly than their physical comorbidities, were unmeasurable. In general, in disaster medicine research, it is difficult to investigate patient morbidity and provide various forms of disaster-related support simultaneously under chaotic circumstances. To prepare for future massive disasters, it is necessary to determine feasible, reliable, and easy-to-use information registry methods that are both practical and allow for statistical analysis.

Despite these limitations, our study revealed that a longer evacuation distance was associated with lower mortality of the evacuated psychiatric hospital inpatients. Patients’ tolerance for longer evacuation transportation and post-evacuation care should be considered when determining appropriate destination hospitals in order to minimize the risk of evacuation-related death. Feasible strategies and continuous training of disaster medicine staff on adequate triage and information-sharing are necessary.

## Supplementary Information

Supplementary Figure 1.

Supplementary Legend.

## Data Availability

The data that support the findings of this study are available from Fukushima prefectural government, but restrictions apply to the availability of these data, which were used under license for the current study, and so are not publicly available. Data are however available from the authors upon reasonable request and with permission of Fukushima prefectural government.
