# Supplementary figures and images for: Long-term observation of mortality among inpatients evacuated from psychiatric hospitals in Fukushima prefecture following the Fukushima nuclear disaster
Source: Sci Rep. 2021 Jul 19;11:14651. doi: 10.1038/s41598-021-94152-1 (PMC8289941; doi:10.1038/s41598-021-94152-1)

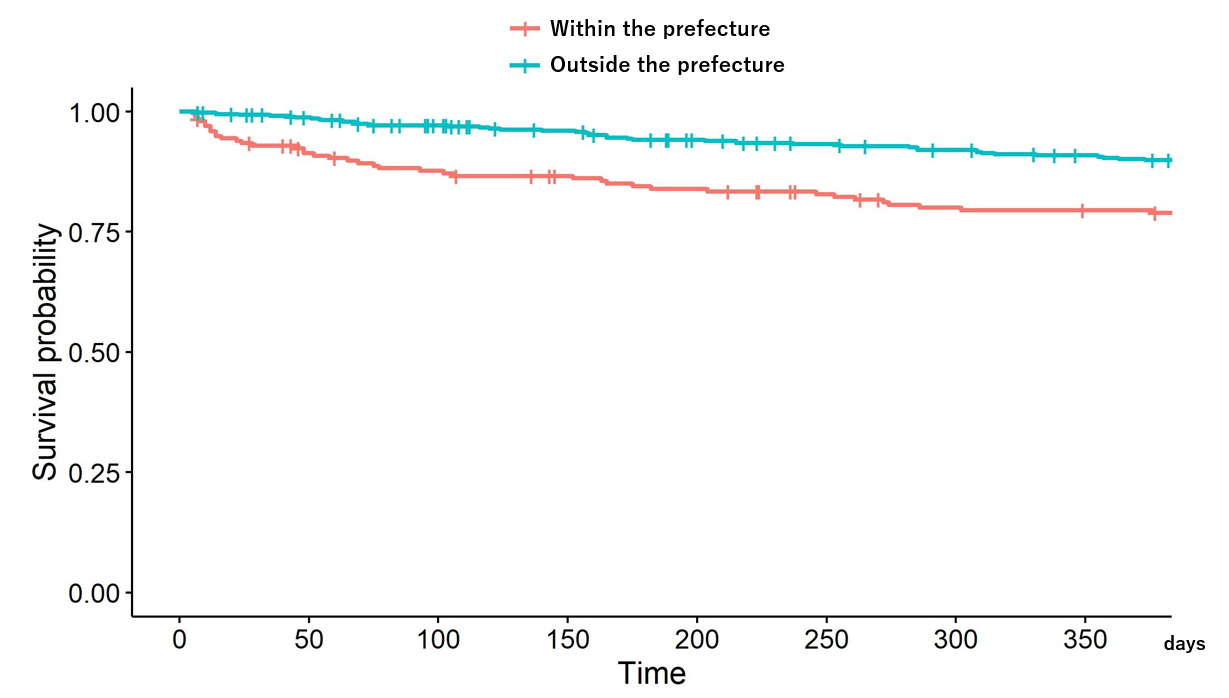

Supplement: Supplementary file 1 — Supplementary Figure 1. [file 41598_2021_94152_MOESM1_ESM.tiff]
