# Supplementary material for: Long-term observation of mortality among inpatients evacuated from psychiatric hospitals in Fukushima prefecture following the Fukushima nuclear disaster
Source: Sci Rep. 2021 Jul 19;11:14651. doi: 10.1038/s41598-021-94152-1 (PMC8289941; doi:10.1038/s41598-021-94152-1)
Supplement: Supplementary file 2 — Supplementary Legend. [file 41598_2021_94152_MOESM2_ESM.docx]

Supplementary Figure 1. Survival probability curves among psychiatric inpatients (one year after March 11, 2011).
